# Auranofin, an Anti-Rheumatic Gold Compound, Modulates Apoptosis by Elevating the Intracellular Calcium Concentration ([Ca^2+^]_i_) in MCF-7 Breast Cancer Cells

**DOI:** 10.3390/cancers6042243

**Published:** 2014-11-06

**Authors:** Elizabeth Varghese, Dietrich Büsselberg

**Affiliations:** Weil Cornell Medical College in Qatar, Qatar Foundation-Education City, P.O. Box 24144 Doha, Qatar; E-Mail: elv2007@qatar-med.cornell.edu

**Keywords:** intracellular calcium, Auranofin, apoptosis, cytotoxicity, anticancer drugs, breast cancer

## Abstract

Auranofin, a transition metal complex is used for the treatment of rheumatoid arthritis but is also an effective anti-cancer drug. We investigate the effects of Auranofin in inducing cell death by apoptosis and whether these changes are correlated to changes of intracellular calcium concentration ([Ca^2+^]_i_) in breast cancer cells (MCF-7). Cytotoxicity of Auranofin was evaluated using MTS assay and the Trypan blue dye exclusion method. With fluorescent dyes SR-FLICA and 7-AAD apoptotic death and necrotic death were differentiated by Flow cytometry. A concentration dependent decrease in the viability occurred and cells were shifted to the apoptotic phase. Intracellular calcium ([Ca^2+^]_i_) was recorded using florescence microscopy and a calcium sensitive dye (Fluo-4 AM) with a strong negative correlation (*r* = −0.713) to viability. Pharmacological modulators 2-APB (50 μM), Nimodipine (10 μM), Caffeine (10 mM), SKF 96365(20 μM) were used to modify calcium entry and release. Auranofin induced a sustained increase of [Ca^2+^]_i_ in a concentration and time dependent manner. The use of different blockers of calcium channels did not reveal the source for the rise of [Ca^2+^]_i_. Overall, elevation of [Ca^2+^]_i_ by Auranofin might be crucial for triggering Ca^2+^-dependent apoptotic pathways. Therefore, in anti-cancer therapy, modulating [Ca^2+^]_i_ should be considered as a crucial factor for the induction of cell death in cancer cells.

## 1. Introduction

Cancer is a result of uncontrolled cell-proliferation, invasion and destruction of healthy tissue. A defective apoptotic program is characteristic of all cancers. One of the main anticancer treatment strategies is to induce apoptosis in cancer cells. To achieve this goal, metal-based anti-cancer drugs have been developed (e.g., platin- and gold-compounds) [1]. Today, cisplatin is widely used for treatment of a broad spectrum of malignancies [2]. Unfortunately, the use of platinum based drugs is limited by their toxicity. There is significant risk of emerging drug resistance and nephrotoxicity linked to the use of cisplatin [3,4]. Therefore, the search for drugs which have a different mechanism to combat cancer growth and which can evade the resistance mechanism is in progress. Therapeutic value of gold compounds is known since ancient times [1]. While originally gold salts were clinically used for treating rheumatoid arthritis [5] over the past decade, they have emerged as a promising class of cytotoxic agents for anti-cancer therapy. Over the past decades, studies have demonstrated the anti-proliferative activity of Auranofin at very low concentrations [6,7].

The mechanism of antitumor activity of gold compound is “DNA-independent”, and different from those of platinum drugs [8]. Marzano and coworkers reported an induction of apoptosis by Auranofin in cisplatin-resistant human ovarian cancer cells [9]. Thioredoxin reductase a mitochondrial enzyme, which is overexpressed in most cancer cells, was identified as one of the major target for Auranofin [10].

Apoptosis is also regulated by intracellular calcium signaling ([Ca^2+^]_i_) and there are considerable amounts of evidence on the central role of [Ca^2+^]_i_ in proliferation and cell death. An increase in [Ca^2+^]_i_ was shown to induce cell death in HeLa-S3 treated with cisplatin [11]. Generally, an elevation of [Ca^2+^]_i_, which could occur through an influx of Ca^2+^ through Ca^2+^-selective channels at the plasma membrane or by release from intracellular stores, is a key factor to trigger apoptosis. Boehning and coworkers emphasized the role of InsP_3_R-mediated calcium release from the endoplasmic reticulum in inducing apoptosis in response to specific stimuli [12]. A recent study reported that clinically relevant concentration of Auranofin evoked Ca^2+^-response by specifically activating TRPA1 channels expressed in human differentiated neuroblastoma cell lines (nIMR-32). This mechanism is independent of the inhibition of the thioredoxin reductase [13]. Wong and colleagues reported that Auranofin, in addition to inhibiting Protein Kinase C, modulated [Ca^2+^]_i_ by mobilizing calcium from multiple storage sites, in a study done in neutrophils [14]. The present study was aimed to investigate the role of [Ca^2+^]_i_ in the induction of apoptosis by Auranofin in the breast cancer cell line MCF-7.

## 2. Results and Discussion

### 2.1. Auranofin Induced a Concentration Dependent Increase of Cell Death and Apoptosis in MCF-7 Breast Cancer Cells

**Figure 1:**
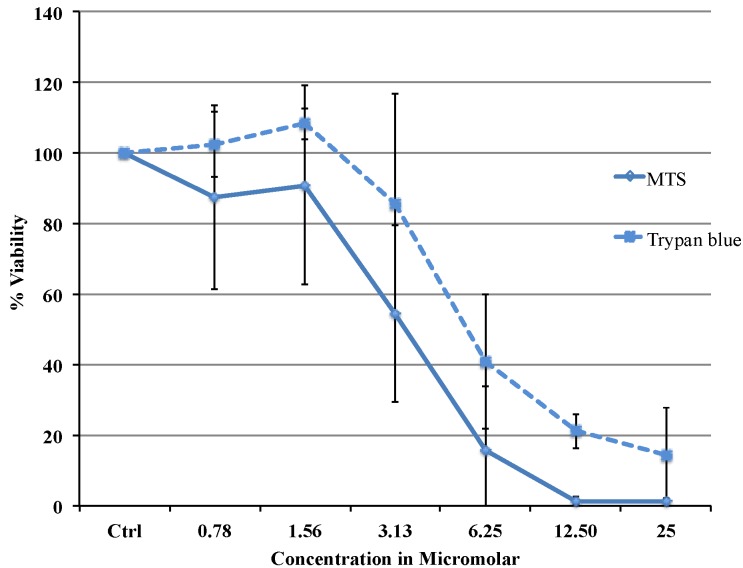
Concentration dependent decrease in viability in cells treated with Auranofin: The effect of Auranofin on the viability of MCF-7 was assessed using MTS assay and trypan blue. Cells were treated with concentration ranging from 0.78 to 25 μM for 24 h. The results were expressed as “%” of the control (control as 100%). The viability dropped significantly for concentration 6.25 μΜ, 12.5 μΜ, and 25 μΜ. Values are the average of 5 replicates and expressed as mean ± SD. Statistical significance between the concentration and viability was determined by Kruskal-wallis test and Bonferroni test was used for multiple comparison (*p* < 0.007). The two methods are not significantly different from each other.

Viability of MCF-7 cells were significantly impaired with increasing concentrations of Auranofin (Figure 1). Concentrations of 0.78 μΜ and 1.56 μΜ had no significant effect on the viability of cells. However, concentrations above 3.12 μΜ resulted in a cytotoxic effect after 24 h of treatment. Viability dropped below 1% in 12.5 μΜ and 25 μΜ. IC_50_ was calculated as 3.37 μΜ. Testing of viability was compared to results obtained using the trypan-blue test. Both methods resulted in a similar trend. After doing multiple comparison (Bonferroni), the control value and the values for low concentrations (0.78 μΜ, 1.56 μΜ) were significantly different (*p* < 0.001) from the data taken with higher concentrations (6.25 μΜ, 12.5 μΜ and 25 μΜ). In addition, a significant difference was found between concentrations of 3.1 μΜ and 12.5 μΜ or 25 μΜ (*p* = 0.002).

**Figure 2:**
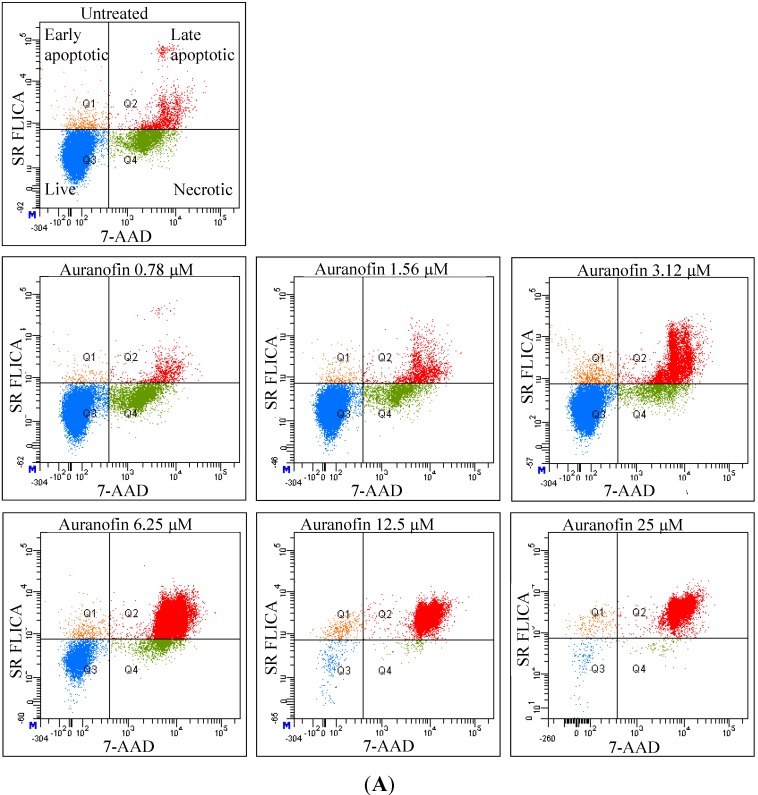

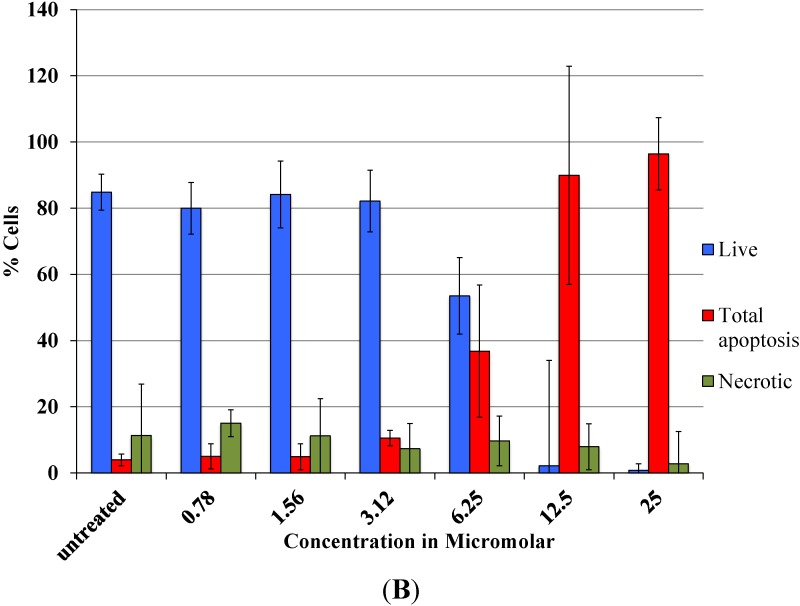
The anti-proliferative effect of Auranofin in MCF-7 breast cancer cell line: (**A**) Auranofin triggers apoptosis: The flow-cytometry dot plot shows 4 populations of cells. Dot plots show the flow cytometry analysis of cytotoxicity using two fluorescent dye SR-FLICA which binds to active caspase enzymes up-regulated for apoptosis and 7-AAD binds to the DNA of membrane-compromised cells. Cells which are negative for both SR FLICA and 7AAD are the viable/live cells. Cells which are positive for both the dyes are the late apoptotic cells. Cells positive only for 7-AAD are the necrotic cells and positive only for SR FLICA are the early apoptotic cells. The panel of images is a representation of a single experiment showing the treated and untreated samples with a clear shift in the type of cells; (**B**) Cell death assessed by two dyes in flow cytometry. The flow cytometry data represents the average of 4 experiments. In the histogram live, apoptotic and necrotic population were plotted against the number of cells. Statistical analysis was carried out with “Non parametric test”. The *p*-value is calculated by Kruskal-Wallis test. The concentrations, 12.5 μM and 25 μM for both the live and apoptotic population were significantly different from the rest of the concentrations. The live (viable) population is completely abolished at the highest concentration.

Increasing concentrations of Auranofin (in a single experiment) resulted in more apoptotic cells (Figure 2A). Under control conditions, the majority of cells were alive (85.6%), some cells were identified to be apoptotic (4 ± 2.9%) or necrotic (11.3 ± 15.5%). This appearance of apoptotic and necrotic cells in the untreated sample is due to normal cell turnover. With increasing concentrations of Auranofin more apoptotic cells occurred and more of the apoptotic cells shifted towards the late apoptotic phase. The late apoptotic cells were 3.2% in the control, but increased to 92.3% in the group treated with 25 μM Auranofin, while percentage of the early apoptotic cells remained unchanged compared to the control. Figure 2B illustrated the histogram of concentration dependent total cytotoxicity (live, apoptotic and necrotic) plotted against the %-population of cells. Early and late apoptotic populations were combined and represented as total apoptosis. With concentrations above 12.5 μM 89.9 ± 10.9%, cells were in the apoptotic phase. The population of the necrotic cells remained below 15% irrespective of the Auranofin concentration (*p* > 0.05). At concentrations of 12.5 μM and 25 μM, the “live” population was found to be almost eliminated. The live population dropped significantly with concentrations higher than 6.25 μM (53.5 ± 31.6%). As it was not possible to do a morphological analysis of the cells by FACS, a clear distinction could not be made between debris and necrotic population which is a limitation of this method, nevertheless considering the total cytotoxicity (apoptosis + necrosis) in the two groups, untreated *versus* e.g., 12.5 μM of Auranofin, the total cytotoxicity was 17% and 97% respectively. A comparison of different concentrations for the different groups is illustrated in Table 1.

The IC_50_ of Auranofin calculated from FACS analysis was 5.1 μM. Pearson’s correlation was tested for the live and apoptotic population and showed a strong reciprocal (*r* = −0.964) link with respect to concentration, which means, the apoptotic population increased proportionally as viability decreased. Hence a concentration dependent increase in apoptotic population was detected.

**Table 1:** Changes of cell populations (“Live”, “Apoptotic” and “Necrotic”) with increasing concentrations of Auranofin as measured by flow cytometry. Values obtained here are the average of 4 independent replicates. The concentrations, 12.5 μM and 25 μM are significantly different from other concentrations (*p* < 0.001). Multiple comparisons represent the significance between different treatment groups. The letter “B” indicates that it is significantly different from the control and other concentrations while “A” indicates that the control and the first 4 concentrations are not significantly different.

Concentration of Auranofin	LiveMean (SD)	ApoptoticMean (SD)	NecroticMean (SD)
Untreated	84.8 (±8.7) ^A^	3.90 (±2.9) ^A^	13.52 (±9.4)
0.78 μM	78.28 (±13.9) ^A^	6.50 (±3.8) ^A^	15.20 (±11.2)
1.56 μM	84.17 (±10.1) ^A^	4.50 (±2.5) ^A^	11.20 (±8.4)
3.12 μM	82.15 (±8.7) ^A^	10.52 (±6.6) ^A^	7.30 (±4.2)
6.25 μM	53.47 (±31.6) ^A^	36.80 (±33.5) ^A^	9.60 (±7.1)
12.50 μM	2.15 (±1.9) ^B^	89.95 (±10.9) ^B^	7.90 (±9.7)
25 μM	0.8 (±0.7) ^B^	96.42 (±3.7) ^B^	2.75 (±3.0)
*p*-value (kruskal-wallis)	0.001	0.001	0.277

The letters represent statistical significance on Bonferroni multiple comparison test (see legend of the table).

### 2.2. Auranofin Induced a Concentration and Time Dependent Elevation of [Ca^2+^]_i_

**Figure 3:**
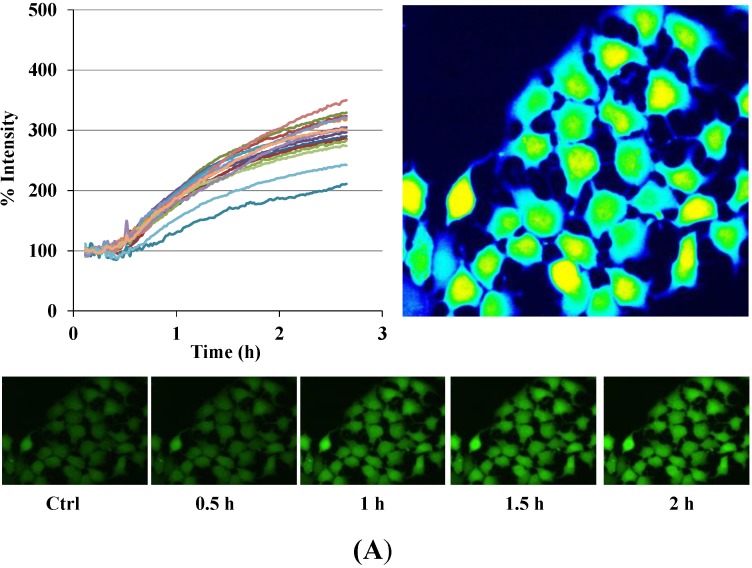

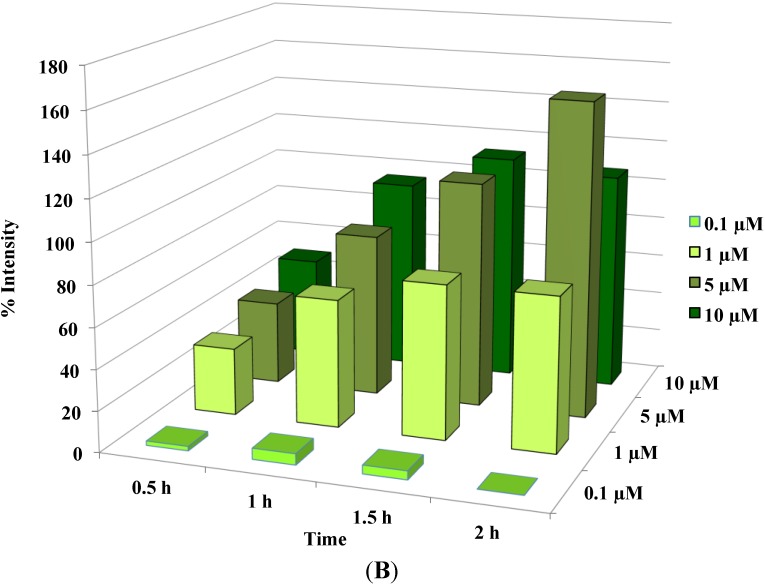
Disruption of [Ca^2+^]_i_ in MCF-7 cells following treatment with Auranofin: (**A**) Auranofin induces a rise in intracellular calcium in MCF-7 breast cancer cell line: Cells loaded with Fluo-4 AM a calcium sensitive dye, with the application of Auranofin showed a time dependent increase in intracellular calcium [Ca^2+^]_i_. The rise of [Ca^2+^]_i_ over time is demonstrated by increase in the fluorescence intensity after application of the drug. The histogram represents the intracellular calcium level of cells selected, from the start of the experiment followed by a gradual increase with the application of the drug. The false color image represents the varying intensities corresponding to calcium concentration in different cells. The lower panel shows images corresponding to different time points. Note: Auranofin was applied continuously for all calcium imaging experiments; (**B**) Auranofin induced a concentration and time dependent elevation in [Ca^2+^]_i_. Time and concentration dependent elevation in [Ca^2+^]_i_ was recorded with 4 different concentrations (0.1, 1, 5, 10 μM). The calcium imaging experiments with Fluo-4 were repeated at least four times for each concentration and the values were averaged. Each concentration was plotted against time and percentage increase in intensity. The diagram shows a very clear time and concentration dependent increase of intracellular calcium. The statistical significance was calculated with Students t-test. All the concentrations were compared to control, concentrations above 0.1 μM showed significant increase in [Ca^2+^]_i_. Mean ± SD: 0.1 μM: (2 ± 8.89) 0.5 h, (5.45 ± 11.39) 1 h, (4.27 ± 16.61) 1.5 h, (4.89 ± 15.73) 2 h. 1 μM: (33.12 ± 22.73) 0.5 h, (62.92 ± 31.53) 1 h, (75.70 ± 22.37) 1.5 h, (75.59 ± 0.72) 2 h, 5 μM: (41.11 ± 27.39) 0.5 h, (80.52 ± 51.77) 1 h, (111.43 ± 70.02) 1.5 h, (154.92 ± 82.51) 2 h. 10 μM: (51.73 ± 20.47) 0.5 h, (97.08 ± 29.31) 1 h, (114.49 ± 25.09) 1.5 h, (107.18) 2 h.

Auranofin triggered increase of [Ca^2+^]_i_ (Figure 3A). The upper left panel represents the increase in fluorescence corresponding to [Ca^2+^]_i_ in single cells. Under control conditions, cells had little difference in their basal [Ca^2+^]_i_ level and a basal level of [Ca^2+^]_i_ was attained in 30 min. After drug application, an increase was recorded which gradually increased during the application of the drug and a steady state was reached after about two hours. Overall, the application of Auranofin resulted in a time and concentration dependent increase of [Ca^2+^]_i_ (Figure 3B) in more than 90% of the cells when the Auranofin concentration was higher than 0.1 μM. Concentrations of 0.1 μM or lower did not alter [Ca^2+^]_i_ significantly till the end of the experiment, while higher concentrations (1, 5 and 10 μM) of Auranofin showed a significant increase in [Ca^2+^]_i_ for the last three concentration within two hours as compared to initial [Ca^2+^]_i_ (0.1 μM: *p* > 0.05; 1 μM: *p* < 0.001; 5 μM: *p* < 0.01; 10 μM: *p* < 0.001).

### 2.3. Cell Death Induced by Auranofin Correlates with the Elevation of [Ca^2+^]_i_

**Figure 4:**
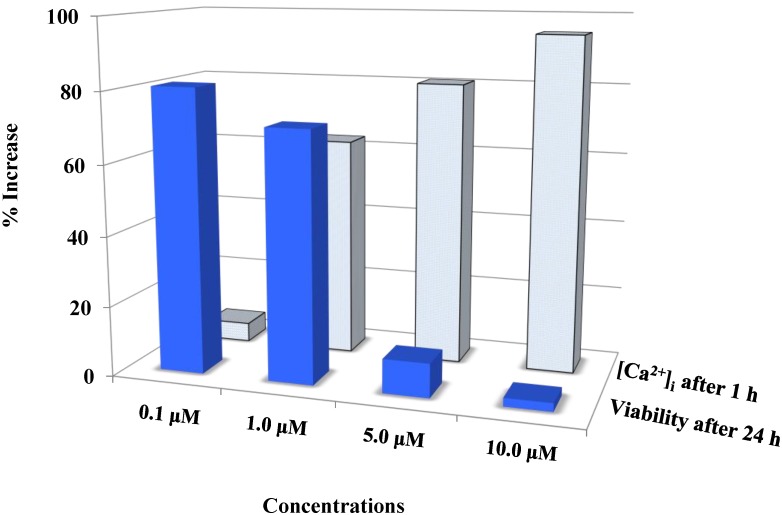
Auranofin induced cell death with the rise in intracellular calcium: The two data sets obtained from calcium imaging experiments and MTS assay were compared using Pearson’s correlation coefficient (2 tailed). Where, r = −0.731 and *p* = 0.005 (** correlation is significant at the 0.01 level). The comparison shows that viability and intracellular calcium have a strong negative correlation. This shows that an increase in [Ca^2+^]_i_ occurred preceding the cell death. 0 < r < 0.3 weak correlation; 0.3 < r < 0.7 moderate correlation; r > 0.7 strong correlation.

The basal calcium level is a key factor in the induction of cytotoxicity. Combining the results from the [Ca^2+^]_i_ imaging experiments and the viability (MTS) assay, showed that the degree with which the viability was decreasing is closely related to increase in [Ca^2+^]_i_ (Figure 4).

### 2.4. Co-Application of Different Calcium Modulators Did not Change the [Ca^2+^]_i_ Levels Significantly

**Figure 5:**
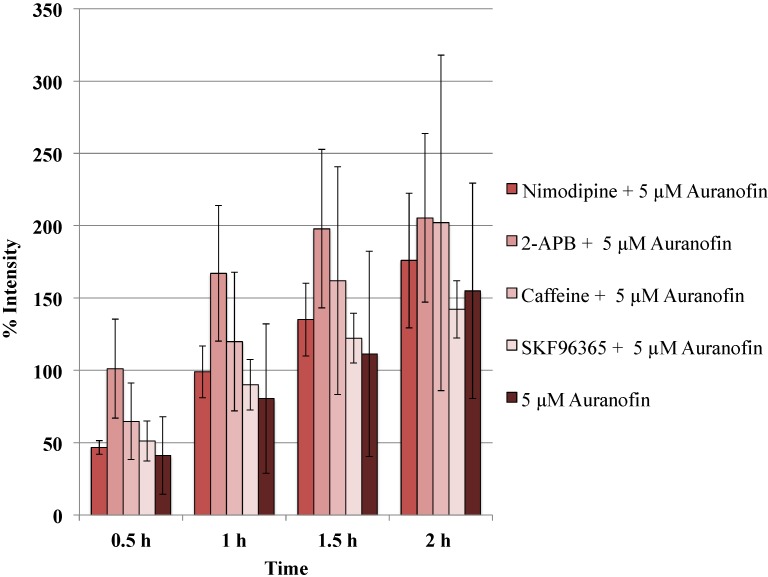
Blockers did not alter the action of Auranofin: Neither of the calcium channel modulators nimodipine 10 μM (L-type Blocker), 2-APB 50 μM (inhibits store operated calcium release) or caffeine (store release), SKF 96365 20 μM (inhibits store operated calcium release) change the effect of Auranofin significantly (*p* > 0.05).

**Figure 6:**
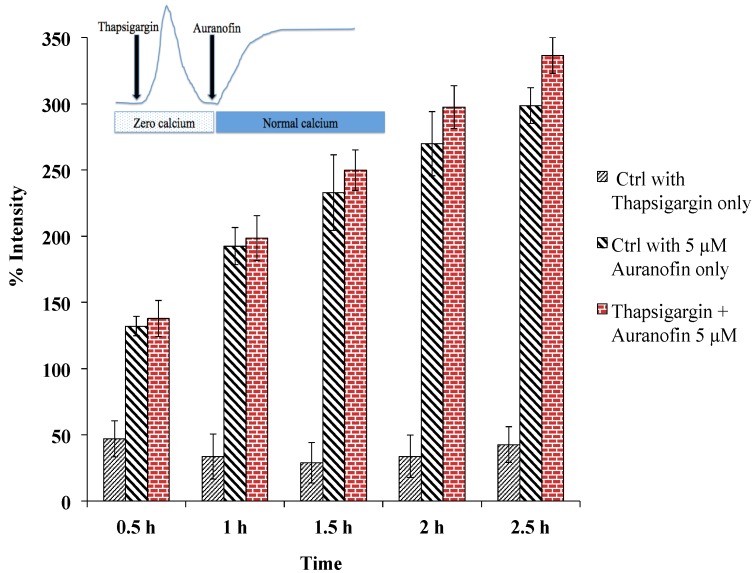
Rise of [Ca^2+^]_i_ does not depend on SERCA, as pre-treatment with thapsigargin (under zero calcium condition followed by application of drug in normal buffer) did not alter the effect of Auranofin induced [Ca^2+^]_i_ rise. Insert shows application protocol.

The use of different [Ca^2+^]_i_-modulators (2-APB (50 µM), caffeine (10 mM), nimodipine (10 μM) caffeine (10 mM) and SKF 96365 (20 μM)) did not change [Ca^2+^]_i_ significantly (Figure 5) over the course of the experiment. This could be an indication that either the time of application has been too short or [Ca^2+^]_i_ is regulated by a more complex modulation. To determine the role of ER calcium in the induction of [Ca^2+^]_i_ rise SERCA was blocked using thapsigargin (1 μM). Thapsigargin did not change the effect caused by Auranofin significantly. The rise of [Ca^2+^]_i_ was significantly different compared to the drug free condition (Control with thapsigargin only). *p* < 0.05 for all time points. SERCA was found to play no major role in Auranofin induced [Ca^2+^]_i_ rise (Figure 6).

## 3. Discussion

Auranofin is a clinically established gold(I) based anti-arthritic drug. Here we examine its ability to induce cell death in a human breast cancer cell-line (MCF-7) and whether its cytotoxicity is related to a drug induced modulation of [Ca^2+^]_i_.

Viability data from MTS assay were very similar to the results obtained with flow cytometry. Cytotoxicity studies show a concentration dependent decrease in viability in the range between 0.78 and 25 µM with an IC_50_ of 3.37 μΜ (MTS) or 5.08 μΜ (flow cytometry data). The difference in IC_50_ obtained from two different assays is most likely due to the different markers as the MTS assay uses mitochondrial enzyme where as FACS method uses the caspase activity. Comparing our cytotoxicity data with the other published data, recently, Liu C Fau-Liu and co-workers reported slightly higher IC_50_ value of 11.4 μΜ in MCF 7 cells [15]. Cytotoxicity of Auranofin for 72 h were tested in a panel of cancer cell-lines by Valentina Gandin and coworkers which reported an IC_50_ of 0.23 μΜ (HL-60), 0.75 μΜ (A549), 0.98 μΜ (MCF-7), 0.34 μΜ (A375), 0.11 μΜ (HCT-15), 0.15 μΜ (HeLa) [16]. Auranofin appears to be 10 times more efficient in inducing cell death than cisplatin in this cell-line where an IC_50_ of 43.7 μΜ was recently reported [17]. Auranofin proves to be more potent than cisplatin to induce cell death in this MCF-7 breast cancer cell line and therefore might be the better choice for treatment.

As revealed by flow cytometry analysis with SR FLICA, cell death is associated with caspase activation. Unfortunately our experiments were not sufficient to prove a direct or indirect involvement of caspase in the induction of cell death. We specifically observed an increase in late apoptotic population with an increase of the drug concentration. The number of cells in the early apoptotic (all treatment) group was not significantly different from the control group. This could be due to the fact that this event could have occurred in a short time interval. Distinct from our study, MCF-7cells treated with 0.25 μg/mL (equivalent to a concentration of about 0.4 μM) of doxorubicin showed a 49-fold increase in early apoptosis (stained with FITC-annexin V and PI) compared to control [18].

A significant shift of the “viable” and “apoptotic” population of cells were observed with the increasing concentration of drug, while the “necrotic” population was not affected, which is a desirable characteristic expected in a potential anticancer drug. This finding indicates that Auranofin interferes with apoptotic pathways but less with the induction of necrosis. This could be an advantage for the use of Auranofin as cell death by necrosis additionally releases multiple inflammatory mediators which could be detrimental for the specific induction of cytotoxicity in cancer cell.

The cellular mechanisms behind triggering cell death in cancer cells could be multifactorial. Anticancer drugs target multiple sites as they can act by binding to DNA and stop replication, they also act by stopping mitosis, or can trigger apoptosis by extrinsic or intrinsic pathways [3].

Anti-proliferative effects of Auranofin on other cancer cell lines were reviewed. Auranofin demonstrated to be potent *in vitro* cytotoxicity against both B16 melanoma cells and P388 leukemia cells and *in vivo* anti-tumor activity against P388 leukemia in mice [7]. Liu C. and coworkers reported the involvement of ROS dependent pathways targeting the mitochondrial dysfunction [15]. As for this study Auranofin was co-applied with selenosystine, the authors conclude that both drugs have synergistic effects on each other. A previous study in A2780 human ovarian carcinoma cells showed that Auranofin triggers caspase 3 activation and induces apoptosis [19]. Auranofin induced a significant level of cell cycle arrest at G1 phase and subsequent apoptosis of myeloma cells [20]. Another report showed that Auranofin decreases the expression of proteasome subunits [21]. These proteins are responsible for protein degradation. A recent study revealed glutathione S-transferase P1-1 (GST P1-1) as a target for Auranofin [22]. Furthermore, the expression of this protein was altered in several cancer cell-lines and it was linked to drug resistance [23]. These and other studies point to numerous intracellular targets of Auranofin.

As calcium is an important signaling ion, which exerts effects on many enzyme and protein functions, it is likely that [Ca^2+^]_i_, also plays a vital role in modulating pathways involving any of the above mentioned targets. Multiple studies support the central role for [Ca^2+^]_i_ in the initiation of apoptosis in cancer cells. Splettstoesser and coworkers have reported that cisplatin induces concentration dependent [Ca^2+^]_i_ by IP_3_-receptor mediated pathway in HeLa-S3 [11]. A study by Günes and coworkers highlighted that a sustained [Ca^2+^]_i_ elevation resulted in an increase of cytotoxicity and apoptosis in neuroblastoma cells treated with cisplatin and/or arsenic trioxide [24]. Cytosolic Ca^2+^ shift was regarded as an earlier marker of cytotoxicity in a study conducted with a panel of toxins tested in different mammalian cell lines [25]. Our study supports the idea that [Ca^2+^]_i_ is modulated by Auranofin, as the strong reciprocal relation of “[Ca^2+^]_i_” *versus* “viability” indicates an important role of [Ca^2+^]_i_ in inducing apoptosis.

The well-established anticancer drug cisplatin targets multiple intracellular sites resulting in cell death and calcium signaling is one among them [26]. Previous studies describe that cisplatin opens a Ca^2+^-conductance at the cell membrane [11], whereas arsenic trioxide releases Ca^2+^ from intracellular stores [27]. Both mechanisms were independent of each other and a co-application of both drugs resulted in a further increase of [Ca^2+^]_i_ and cell death [24]. Preliminary data from our lab were compared with our cisplatin treatment group and both compounds were found to modulate calcium in MCF-7 breast cancer cell-line [28,29]. The authors on a study using promyelocytic leukemia cells (HL-60), stated that it is not just the elevation of [Ca^2+^]_i_ but the additional release of calcium from TG-sensitive calcium stores of the endoplasmic reticulum that triggers apoptosis [30]. Only a few publications focused on the intracellular calcium modifications caused by Auranofin. A similar study done in neutrophils by Wong and coworkers reported a pleotropic mode of action of the drug. They found that Auranofin initially induced a slow release of calcium from the stores followed by a delayed influx of extracellular calcium showing a biphasic effect. They also emphasized an indirect role of Protein kinase C in modifying [Ca^2+^]_i_ [14].

With our analysis, we could not conclusively answer the question whether Auranofin induced apoptosis was a direct effect of the rise of intracellular calcium or an indirect effect and whether the [Ca^2+^]_i_ elevation directly caused an activation of caspases. Our studies with the blockers did not reveal the exact mechanism of [Ca^2+^]_i_ elevation. Taking into consideration the complexity of Ca^2+^ signaling, at this juncture it remains difficult to elucidate the exact mechanism behind the apoptosis induced by elevation of [Ca^2+^]_i._ In addition the amount of calcium released from the ER or the mitochondria and the calcium entry through the plasma membrane for the observed elevations in [Ca^2+^]_i_ needs further investigation in order to distinguish whether their functions are interconnected.

## 4. Experimental

### 4.1. Cell Culture

Human MCF-7 breast cancer cells were purchased from the American Type Culture Collection (Cat No. HTB-22, Manassas, VA, USA). Cells were grown in DMEM (Sigma, Virginia Beach, VA, USA) with 10% FBS (Sigma) and incubated at 37 °C in a humidified incubator containing 5% carbon dioxide.

### 4.2. Viability Test

Cytotoxic effect of Auranofin was evaluated by MTS assay (CellTiter 96(R) Aqueous One Solution from Promega, Madison, WI, USA). Untreated cells were taken as control and a range of concentrations from 0.78 to 100 μM were tested. MTS (3-(4,5-dimethylthiazol-2-yl)-5-(3-carboxymethoxy-phenyl)-2-(4-sulfophenyl)-2H-tetrazolium) in the presence of phenazine methosulfate (PMS), produces a formazan product that has an absorbance maximum at 490–500 nm in aqueous solution.

MCF-7 cells for the MTS assay were plated in 96 well plates. Phenol red free DMEM with 10% Foetal Bovine Serum was used throughout the procedure. 20,000 cells were plated in each well. The cells were left at 37 °C in a CO_2_ incubator overnight for equilibration and on the second day various dilutions of the drug were added. The cells were treated with the drug for 24 h and on the third day the plate was washed with saline and the MTS reagent was added. The absorbance was taken at 492 nm using an Envision spectrophotometer plate reader. The cell viability was expressed as percentage of viable cells compared to untreated control using the equation: (% Viable = Absorbance_test_/Absorbance_control_ × 100). Untreated control was considered as 100% viable. IC_50_ was defined as the drug concentration that reduced the number of living cells by 50%. Trypan blue dye exclusion test was carried out with TC20 Automated cell counter (Bio-Rad, Hercules, CA, USA).

### 4.3. Flow Cytometry

Total cytotoxicity and apoptosis were assessed using a kit with two fluorescent dyes SR-FLICA and 7-AAD (immunochemistry technologies, Bloomington, MN, USA). The former permeates through the intact plasma membrane and binds covalently to active caspases which initiates a positive feed-back signal for apoptosis and the latter binds to DNA but does not pass through an intact cell membrane and therefore can only bind to the DNA of the compromised cell membrane. Four types of cell population were quantified: (1) early apoptotic; (2) late apoptotic; (3) live and (4) necrotic. SR-FLICA stains the apoptotic cells and 7-AAD binds to the necrotic/dead cells. Cells were treated with the drug for 24 h and harvested. The cells were trypsinized; the cell concentration was determined and adjusted to 1 × 10^6^ cells/mL. The cells were first incubated with SR-FLICA at 37 °C for 45 min followed by 7-AAD for 10 min in the ice. After the incubation, the cells were washed twice in PBS and re-suspended in 400 μL FACS buffer supplied along with the kit. The fluorescence was acquired using BD LSR Fortessa with 488 nM laser for excitation and emission with 2 filters 585/15 and 610/20 for SR-FLICA and 7-AAD respectively. Data was processed using FACSDiva 6.3. The non-aggregated single cells were selected through SSC (Area, Height, Width) and FSC (Area, Height, Width). PMT was defined on untreated unstained cells. Single stained samples were used for compensation and for gating. 50,000 events were recorded for each sample. Cells were sorted in four different types of cell population (indicated by four quadrants (Q) in the graph). Cells which are negative for both dyes are viable cells (Q3). The cells positive only for SR FLICA were early apoptotic (Q1) and that which were positive only for 7-AAD were necrotic (Q4). The cells positive for both dyes were considered late apoptotic cells (Q2). The double stained cells were analyzed offline in excel. The early and late apoptotic cells were combined and represented as total apoptosis. The results were illustrated as dot plots with the fluorescence corresponding to SR FLICA and 7-AAD plotted against x and y-axis and as histogram where, fluorescence was plotted against cell count.

### 4.4. Calcium Imaging

Cells were plated in a 35 mm “eazygrip” BD Falcon tissue culture plates (Franklin Lakes, NJ, USA). The plates were washed with Tyrode’s buffer (145 mM NaCl, 2.5 mM KCl, 10 mM HEPES, 10 mM Glucose, pH 7.4 by 1 M NaOH, 1.5 mM CaCl_2_ and 1.2 mM MgCl_2_) and incubated with the fluorescent dye Fluo-4 AM (Molecular Probes, Eugene, OR, USA) a calcium sensitive dye. These calcium indicators exhibit an increase in fluorescence upon binding Ca^2+^. The AM ester is colorless and non-fluorescent until hydrolyzed. The cells were loaded with Fluo-4 AM for 40 min and washed, incubated for an additional 15 min to complete the de-esterification process of the dye. Fluorescent images were taken with Olympus Microscope BX51 Wi with Xenon Arc Burner and “Xcellence rt” software (Olympus soft imaging solutions GMBH, München, Germany). A water immersion objective (40×) was used for recording. An excitation wavelength of 494 nm and an emission wavelength of 516 nm were used. Fluorescent images were taken once a minute for up to 3.5 h. The buffer in the dish was exchanged through a continuous flow system (2 mL/min). Auranofin was applied when cells attained a stable basal [Ca^2+^]_i_ level for at least 30 min (control). The baseline for [Ca^2+^]_i_ was obtained by averaging 20–25 frames at the beginning of the experiment (before drug application) and was considered as 100%. For each experiment at least 15–25 cells were recorded. Results were analyzed offline. For each experiment the Regions of Interest (ROI) were selected and only cells which were intact over the entire experiment were selected for analysis. Data (averaging the intensity of the fluorescent in the ROI) were exported to excel. Background values corresponding to each image were subtracted. The values obtained from the control conditions were averaged and set as control value (100%) to calculate the percentage change in intensity over time by the equation = (F/F_0_) × 100. Where, F is the intensity at a given time and F_0_ is the average intensity of the control window. The results were represented as 3D graph with time and concentration plotted against percentage increase in [Ca^2+^]_i_. Images were represented in “rainbow” scale (false color) to highlight the difference in fluorescent intensities. Blue color represents lowest [Ca^2+^]_i_ and yellow-red shows regions of high [Ca^2+^]_i_).

### 4.5. Pharmacological Manipulations

The following calcium channel modulators were used (under normal calcium external concentration) to identify which mechanisms might be involved in the changes of [Ca^2+^]_i_: Caffeine (10 mM) for emptying the ER stores, 2-APB (50 μM) for inhibiting store-operated calcium (SOCE) release and a IP_3_-receptor blocker [31,32], SKF 96365 (20 μM) for inhibiting store operated calcium release. Nimodipine (10 µM), a di-hydropyridine calcium channel blocker, to block the calcium entry through L-type channels (Tocris, Ascent Scientific, Bristol, UK). All of these substances were pre-applied before Auranofin and simultaneously with the anti-cancer drug. Pre-application of Thapsigargin (1 µM) was done under zero calcium condition followed by the application of drug in normal calcium containing buffer.

### 4.6. Data Analysis

Each experiment was replicated three to five times and the mean ± SD was calculated. Data were statistically analyzed using IBM-SPSS version 20 (IBM, Armonk, NY, USA) as well as Microsoft excel (2011) (Microsoft Corporation, WA, USA). To test the association between concentration and viability of the cells (MTS and Trypan blue assay) means and standard deviations were computed and the nonparametric Kruskal-Wallis test was used to test for the associations. To test the association between the concentrations and the different populations of cells (flow cytometry), we used the same methods of analysis as MTS Assay.

Post hoc analysis using Bonferroni correction (*p* < 0.007) was applied for the multiple comparisons in the different concentration groups. The letter represents whether there was a statistical significance on the Bonferroni multiple comparison test. *t*-Test was done for calcium imaging experiments. Pearson’s correlation was applied for comparing viability (live cells) to [Ca^2+^]_i_ and viable cells to apoptotic cells. Differences were considered significant when p-values were smaller than 0.05.

## 5. Conclusions

Results indicate that the Auranofin induced an elevation of [Ca^2+^]_i_ in MCF-7 cell line. Prolonged elevation of calcium might be a key factor for triggering a Ca^2+^-dependent apoptotic pathway. To the best of our knowledge, this is the first study which analyzes the modulation of [Ca^2+^]_i_ by Auranofin in a breast cancer cell line.

We conclude that modulating [Ca^2+^]_i_ is a crucial factor to induce cell death in cancer cells. An answer to the mechanism of calcium modulation by Auranofin, by studying the calcium channels and pumps will help to improve the efficiency of drug action. To understand the exact mechanisms of [Ca^2+^]_i_ modulation, further experiments are needed.
